# Fine Mapping of the Wheat Leaf Rust Resistance Gene *LrLC10* (*Lr13*) and Validation of Its Co-segregation Markers

**DOI:** 10.3389/fpls.2020.00470

**Published:** 2020-05-06

**Authors:** Lina Qiu, Huifang Wang, Yinghui Li, Weidong Wang, Yujia Liu, Junyi Mu, Miaomiao Geng, Weilong Guo, Zhaorong Hu, Jun Ma, Qixin Sun, Chaojie Xie

**Affiliations:** ^1^State Key Laboratory for Agrobiotechnology, Key Laboratory of Crop Heterosis and Utilization (MOE), Key Laboratory of Crop Genetic Improvement, China Agricultural University, Beijing, China; ^2^Institute of Evolution, University of Haifa, Haifa, Israel; ^3^College of Agronomy Hebei Agricultural University, Hebei Agricultural University, Baoding, China

**Keywords:** wheat, *LrLC10* (*Lr13*), leaf rust resistance, fine mapping, marker-assisted selection (MAS)

## Abstract

Wheat leaf rust, caused by the fungus *Puccinia triticina* Eriks. (*Pt*), is a destructive disease found throughout common wheat production areas worldwide. At its adult stage, wheat cultivar Liaochun10 is resistant to leaf rust and the gene for that resistance has been mapped on chromosome 2BS. It was designated *LrLC10* and is the same gene as cataloged gene *Lr13* by pedigree analysis and allelism test. We fine-mapped it using recessive class analysis (RCA) of the homozygous susceptible F_2_ plants derived from crosses using Liaochun10 as the resistant, male parent. Taking advantage of the re-sequencing data of Liaochun10 and its counterpart susceptible parent, we converted nucleotide polymorphisms in the *LrLC10* interval between the resistant and susceptible parents into molecular markers to saturate the *LrLC10* genetic linkage map. Four indel markers were added in the 1.65 cM map of *LrLC10* flanked by markers *CAUT163* and *Lseq22*. Thirty-two recombinants were identified by those two markers from the 984 F_2_ homozygous susceptible plants and were further genotyped with additional ten markers. *LrLC10* was finally placed in a 314.3 kb region on the Chinese Spring reference sequence (RefSeq v1.0) that contains three high confidence genes: *TraesCS2B01G182800*, *TraesCS2B01G182900*, and *TraesCS2B01G183000.* Sequence analysis showed several variations in *TraesCS2B01G182800* and *TraesCS2B01G183000* between resistant and susceptible parents. One KASP marker and an indel marker were designed based on the differences in those two genes, respectively, and were validated to be diagnostic co-segregating markers for *LrLC10*. Our results both improve marker-assisted selection and help with the map-based cloning of *LrLC10*.

## Introduction

Globally, common wheat (*Triticum aestivum*) is one of the most commonly cultivated crops, comprising 20% of human caloric intake and 15% of cultivated area in the world (FAOSTAT, 2015; WAP, 2017). Wheat leaf rust, caused by *Puccinia triticina* Eriks. (*Pt*) is one of the most damaging diseases of wheat, especially in coastal regions or areas with high temperatures and humidity during the wheat maturing seasons (Kolmer et al., 2018). In China, past widespread wheat leaf rust epidemics have caused severe yield losses (Dong, 2001; Zhou et al., 2013). In the future, due to impending climate change, leaf rust is expected to damage wheat production even more (Jiang et al., 2018). Utilization of wheat resistant cultivars considered a most effective, economical and environmentally-friendly strategy for controlling this disease (Bariana et al., 2007; Singh et al., 2013).

Currently, about 80 leaf rust resistant genes have been reported and formally named in common wheat or its relatives (McIntosh et al., 2017; Qureshi et al., 2018), and by using different types of molecular markers, most of these genes have been mapped on the wheat chromosomes^1^. Development of robust molecular markers linked to resistance genes is essential in wheat disease resistance breeding, especially for resistance gene pyramiding. Nevertheless, among these designated leaf rust resistance genes, only a few have tightly linked molecular markers for marker-assisted selection^2^.

Because of limited wheat genomic sequence data, developing molecular markers for wheat genes has been difficult. But now, by combining the *T. aestivum* ‘Chinese Spring’ (CS) IWGSC RefSeq v1.0 genome^3^ with annotations of high-quality gene models, these difficulties have been reduced, especially for gene location and markers development (Clavijo et al., 2017). Discovery of the highly abundant, locus-specific wheat nucleotide variations that can be used to identify the relevant genes are now within grasp because of affordable next-generation sequencing (Varshney et al., 2014; Xu et al., 2017). Compared with other kinds of markers, kompetitive allele-specific PCR (KASP) assays accelerate the conversion of DNA variations into available gene-linked markers. Taking advantage of such whole genomic sequences, Wu et al. (2018b) mapped wheat yellow rust resistance gene *Yr26* on a 0.003-cM interval on chromosome 1B near the centromere, Narang et al. (2019) defined wheat leaf rust resistance gene *LrP* and yellow rust resistance gene *YrP* on a 15.71 Mb region on 5DS in the CS RefSeq v1.0 genome assembly, and Wu et al. (2019) localized the *Pm52* locus within a 5.6 Mb interval on the long arm of chromosome 2B (2BL).

Bulked segregant analysis (BSA) can rapidly identify markers linked to target genes (Michelmore et al., 1991), and it has been improved by bulking homozygous recessive plants and using recessive class analysis (RCA) to map specific genes (Zhang et al., 1994). RCA is highly efficient, with a lower probability of misclassification and more reliability than using a random F_2_ population. Furthermore, this approach avoids creating F_2__:__3_ families and screening the entire F_2_ population, thus saving time in fine mapping and map-based cloning. RCA has been proven to map genes efficiently and reliably (Zhang et al., 1994; Yao et al., 1997; Mei et al., 1999; Chen et al., 2006; Kiswara et al., 2014). In wheat, rapid gene mapping using RCA has been used to map a sterile female gene (Dou et al., 2009) and a stripe rust resistance gene, *YrLM168a* (Feng et al., 2015), and to successfully map and clone the powdery mildew resistance gene *Pm60* (Zou et al., 2018).

In wheat, *Lr13*, first identified in the Canadian cultivar “Manitou” in 1966, is an important adult-plant leaf rust resistance gene (McIntosh et al., 1995) that is widely found in wheat cultivars (e.g., ‘Frontana,’ ‘Frondoso,’ and ‘Fronteria’) and used in many breeding programs throughout the world (Roelfs, 1988; Pathan and Park, 2006). In China, *Lr13* is one of the main resistance genes and confers effective resistance to leaf rust (Singh et al., 1999; Yuan et al., 2007; Yuan and Chen, 2011; Ren et al., 2015; Zhang et al., 2019). Previous studies indicated that *Lr13* was located on chromosome 2BS (McIntosh et al., 1995), and Bansal et al. (2008) reported *Lr13* was delimited to a 13.8 cM interval flanked by markers *Xksm58* and *Xstm773b*. Recently, using a segregating population of *Lr13* near-isogenic lines with simple sequence repeat and KASP markers, Zhang et al. (2016) mapped *Lr13* to a small interval of 10.7 cM, and the closest marker was *kwh37* (4.9 cM). A morphological marker, hybrid necrosis gene *Ne2m*, was found linked to *Lr13* by Singh and Gupta (1991), but it cannot be used to accurately detect *Lr13* (Anand et al., 1991). Therefore, a co-segregated and diagnostic marker for *Lr13* in molecular breeding is yet unavailable.

In this study, we confirmed that the leaf rust resistance gene *LrLC10* in Liaochun10 is *Lr13* by pedigree analysis, and finely mapped it to a close interval with recessive class analysis (RCA) through the markers developed according to the re-sequencing data of the parental lines. We also developed molecular markers that were closely linked to *LrLC10* and that can be used to facilitate marker-assisted selection of *LrLC10* in wheat resistance breeding.

## Materials and Methods

### Plant Materials

The spring wheat cultivar Liaochun10, is highly resistant to leaf rust, was crossed with two susceptible wheat lines Han 87-1 (87-1) and 7D49 (a wild emmer wheat introgression line created by the crossing IW123/Zheng98//87-1^∗^2), to construct two F_2_ segregating populations of 3,908 plants. Wild emmer wheat IW123 was donated by T. Fahima and E. Nevo, University of Haifa, Israel and *Lr13*-carrier line RL4031 was provided by Zaifeng Li, College of Plant Protection, Heibei Agricultural University, China. The 1,395 F_2_ plants derived from the Liaochun10 × RL4031 cross were used to test allelism. A total of 35 cultivars with known presence/absence of *Lr13* were chosen to validate the co-segregating markers (Table 1). A panel of 524 Chinese wheat accessions/landraces was used to test the *Lr13* frequencies (Supplementary Table S1). Through all the experiments, a susceptible, common wheat line, Xuezao, was used to check for successful inoculation.

**TABLE 1:** Phenotype of 35 common wheat cultivars and genotyped with *Lseq302* and *Lseq102.*

Accession	Phenotype	*Lseq302*	*Lseq102*	Progressive necrosis
Zhoumai30	Resistant	A	A	N
Apache	Resistant	A	A	Y
Maris Dove	Resistant	A	A	Y
Gaoyou2018	Resistant	A	A	Y
Gaoyou9828	Resistant	A	A	Y
Gaoyou5766	Resistant	A	A	Y
Kenong2009	Resistant	A	A	N
Shannong06-278	Resistant	A	A	N
Cunmai11	Resistant	A	A	N
Yannong999	Resistant	A	A	N
Yunmai53	Resistant	A	A	Y
16Y2N137	Resistant	A	A	Y
16Y2N132	Resistant	A	A	Y
16Y2N1688	Resistant	A	A	Y
Zhoumai36	Resistant	A	A	N
90214	Resistant	A	A	Y
Zhouyuan9369	Resistant	A	A	N
Bainong419	Resistant	A	A	N
Nongda211	Resistant	A	A	Y
Nongda212	Resistant	A	A	Y
Altgold	Resistant	A	A	Y
Liangxing66	Resistant	A	A	N
CI12633	Resistant	A	A	Y
Nongda1108	Resistant	B	B	N
Zhongmai66	Resistant	B	B	N
Nongda3432	Susceptible	B	B	N
Liangxing99	Susceptible	B	B	N
Xinmai26	Susceptible	B	B	N
Shi4185	Susceptible	B	B	N
Nongda4503	Susceptible	B	B	N
Jimai229	Susceptible	B	B	N
Xuezao	Susceptible	B	B	N
Gao5	Susceptible	B	B	N
CA1062	Susceptible	B	B	N
Nongda3753	Susceptible	B	B	N

*Y, indicates progressive necrosis; N, indicates the cross was not tested; A, indicates the band is identical with resistant parent; B, indicates the band is identical with susceptible parent.*

### Field Evaluation of Leaf Rust Symptoms at the Adult Stage

Wheat leaf rust isolate PHT (provided by the Institute of Plant Protection, Chinese Academy of Agricultural Sciences, Beijing, China) was used as the inoculum. PHT isolate was avirulent on Liaochun10 and RL4031.

The populations were sown at the experiment farm of China Agriculture University, Beijing, China. At the late tillering stage (Feekes stage 5) at least one tiller of each plant was inoculated by injecting urediniospore suspended in 0.1% Tween 20 into the leaf bundle with a 10 mL syringe. The urediniospore was propagated in the greenhouse on the susceptible control, Xuezao.

The infection type of the flag leaf and the top second leaf of each individual was evaluated about 1–2 month post-inoculation when the susceptible control was fully infected, based on an infection type scale of 0–4, where 0 indicated no visible symptoms, 0; indicated hypersensitive flecks, and 1–4, indicated small uredinia with necrosis, small- to medium-sized uredinia with green islands and surrounded by necrosis or chlorosis, medium- to large-sized uredinia with chlorosis, and large uredinia without chlorosis, respectively. Values 0–2 were categorized as resistant and 3–4 were classified as susceptible (Roelfs et al., 1992). A second assessment was conducted for each plant 4 days after the first examination.

### Allelism Tests

We used an F_2_ population derived from Liaochun10 × RL4031 to determine the allelic relationships between genes *Lr13* and *LrLC10*. The responses of each F_2_ plant to *Pt* race PHT was determined by the rust response method described above.

### Development of Molecular Markers

The sequences of all the markers anchored in the *LrLC10* (*Lr13*) genetic linkage map were used as queries to search against the Chinese Spring reference genome sequence (RefSeq v1.0) to define the genome interval of the resistance gene on chromosome arm 2BS. Near the *LrLC10* locus, single-nucleotide polymorphisms (SNPs) or insertion/deletion (indel) polymorphisms were found based on re-sequencing result of the two parents (the concrete method refer to Chai et al., 2018) and the 300 bp flanking sequence of those indel sites which were ≥5 bp that were obtained from the Chinese Spring reference genome sequence^4^, Primer3 (v.0.4.0)^5^ was used to design the indel markers. The SNPs or indels (<5 bp) were converted into kompetitive allele-specific PCR (KASP) markers, which were designed using PolyMarker^6^. The markers used in this study were listed in Table 2.

**TABLE 2:** Markers used in this study.

Marker	Marker type	Forward primer	Reverse primer	Annealing temperature
*Lseq29*	Indel	CGCTTCTATCCTTGGTGG	AGCATTGGAGCACAGAGA	56
*Lseq31*	Indel	CCCAGTCTTGACCAGGTTGA	CTGGCCCTTCGTCCTATCTG	56
*Lseq35*	Indel	ACTCAACAGGCTAATCAGGGT	GAACAACCACTGACATCGGG	56
*Lseq54*	Indel	CCACCAAACAAACTAAAGAAGC	CACCCGATGACGATAAGC	56
*Lseq55*	Indel	CAGTTGGACGAGGGGAGTG	GAACCACAATCCTGCAGCAG	56
*Lseq301*	Indel	ACACTCAATGGGGTCGCATA	AGAACACCGGATTTGCTTGT	56
*Lseq102*	Indel	GGCTTCTTCATCATCAGGTACG	GCATGCGATCCAACCCTTTG	56
*Lseq85*	Indel	CAGCGATGGATGCCGAAATA	CAGCCTACTCCTCCTGCTC	56
*Lseq99*	Indel	TACGACCATTGCCGGATGAT	CTACATGTGGTGCTCGTACG	56
*Lseq100*	Indel	ATTGCGGAGTAGTGCTTTCG	TAATCCTGCAATCACGAGCG	56
*Lseq3*	Indel	CCTCTATGTCACCCGCAAGT	CAGGGTCTCAAGTGGGGAAG	56
*Lseq11*	Indel	CGCTAATGGGCTGGCTTAAC	GTTTCGAACCTGACACGCTG	56
*Lseq22*	Indel	ACGTACAGAGAAGTGCCCAC	GGCTCAAGTGGGTCTCTGAA	56
*Lseq302*	KASP	GAAGGTGACCAAGTTCATGCTGTG GTGTAATTATTGGGCTCATCACA	CGCTCAAGTTGAAGGTTGAGTGCAA	–
		GAAGGTCGGAGTCAACGGATTGTG GTGTAATTATTGGGCTCATCACT		

### Marker Genotyping Assays

PCR amplification was conducted in a 10 μL reaction volume consisting of 5 μL 2 × Tag PCR StarMix with loading dye, 50–100 ng/μL DNA 1.5 μL, 1.5 μL primer (mixture of forward and reverse primer, 2 μM), and 2 μL H_2_O. PCR proceeded with initial denaturation at 94°C for 5 min, then 35 cycles at 94°C for 30 s, 30 s at 50–60°C for primer annealing (depending on the specific primers), 72°C for 30 s of extension; and the final extension at 72°C for 5 min. The PCR product was separated in either 8% or 10% non-denaturing polyacrylamide gels (acrylamide:bisacrylamide = 39:1) that were silver stained and photographed. KASP assays were performed following the protocol described in Wu et al. (2018a).

### Construction of the Genetic Linkage Map

We performed chi-square analysis of the leaf rust test data from the segregating F_2_ populations to confirm the goodness-of-fit of the observed ratios to theoretical expectations. The recombination frequencies of the resistance gene and the markers were calculated according to Chen et al. (2006). Using the Kosambi mapping function, we converted the recombination frequencies to centimorgans (Kosambi, 1943) and drew the genetic map using Mapdraw v2.1 (Liu and Meng, 2003).

## Results

### Genetic Analysis of Wheat Leaf Rust Resistance Gene *LrLC10* in Two Segregating Populations

**FIGURE 1:**
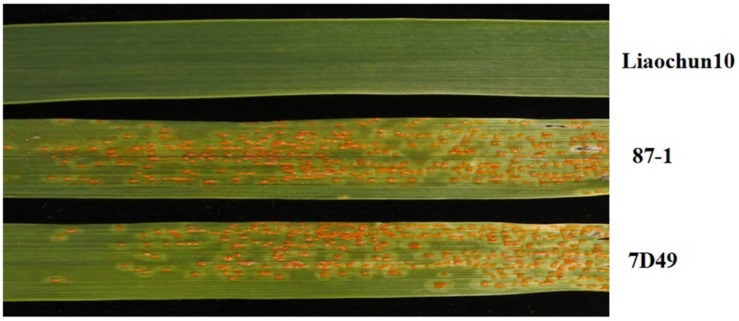
Phenotype of resistant parent Liaochun10, susceptible parent 87-1, and 7D49 30 days after inoculation with *Pt* race PHT.

The parental lines 87-1 and 7D49 were highly susceptible to *Pt* race PHT, [infection type (IT) = 3], whereas Liaochun10 was highly resistant (IT = 0, Figure 1). We examined the two segregating F_2_ populations that grew from crossing the susceptible lines with Liaochun10. The 87-1 crossed with Liaochun10 produced the F_2_ population including 3,057 plants, of which 2,300 were resistant and 757 were susceptible to *Pt* isolate PHT (χ^2^_3__:__1_ = 0.092, *P* > 0.05). In the F_2_ population derived from 7D49 crossed with Liaochun10, 624 plants were resistant and 227 were susceptible to *Pt* race PHT (χ^2^_3__:__1_ = 1.268, *P* > 0.05). These results indicated that leaf rust resistance in Liaochun10 is controlled by a single dominant gene.

### Allelism Test of *LrLC10* and *Lr13*

Liaochun10, RL4031, and the 1,395 F_2_ plants from the cross of Liaochun10 × RL4031 were evaluated against *Pt* race PHT. We found no susceptible plants, thus confirming that *LrLC10* in Liaochun10 was on the same locus as *Lr13*. Since there was *Lr13* donor parents Frontanan and UP301 in the Liaochun10 pedigree (Singh and Gupta, 1991; He et al., 2001; Pathan and Park, 2006), we concluded that leaf rust resistance gene *LrLC10* in Liaochun10 is *Lr13*.

### Molecular Mapping of Leaf Rust Resistance Gene *LrLC10* (*Lr13*)

**FIGURE 2:**
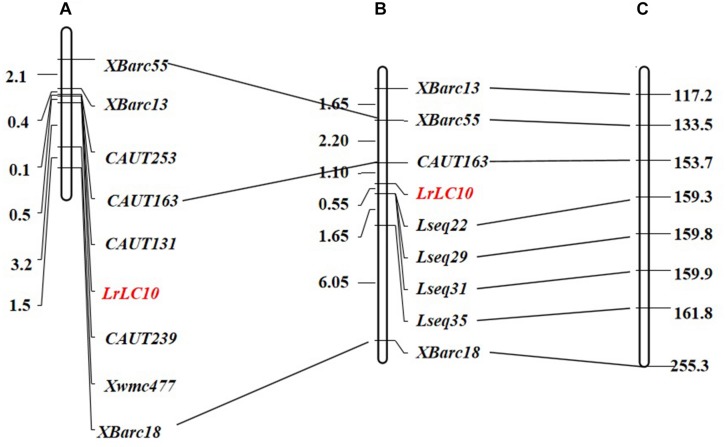
Genetic linkage map of leaf rust resistance gene *LrLC10* and corresponding physical position on the Chinese Spring RefSeq v1.0. **(A)** Lv et al. (2017) genetic map. **(B)** The genetic linkage map of *LrLC10*, genetic distances are shown to the left in cM. **(C)** The corresponding physical location of the polymorphic linkage markers of *LrLC10* on the chromosome 2BS of Chinese Spring RefSeq v1.0, physical distances are shown to the right in Mb.

We chose 92 extremely susceptible individuals from the 7D49 × Liaochun10 F_2_ population to be re-genotyped using markers linked to *LrLC10* that were established by Lv et al. (2017) (Figures 2A,B). To define the *LrLC10* physical interval, we searched the sequences of all markers anchored in the genetic map against the Chinese Spring reference genomic sequence (RefSeq v1.0) and found that the relative physical positions of those markers were generally consistent with the genetic map (Figures 2B,C). Two flanking markers, *CAUT163* and *Xbarc18*, spanned an approximately 100 Mb region (153,676,602–255,348,323) in the reference genome, and here we detected numerous sequence variations between the parents when we analyzed the re-sequencing data. Twenty indel primer pairs were designed based on those insertion/deletion polymorphisms we found between parental lines in the 11 Mb (159,000,000–170,000,000) section that was 6 Mb from marker *CAUT163* going toward *LrLC10*. Among these, 4 markers (*Lseq22*, *Lseq29*, *Lseq31*, and *Lseq35*) were successfully added to the genetic map and the *LrLC10* gene was delimited within a 1.65 cM area between markers *CAUT163* and *Lseq22*, an interval corresponding to a 5.7 Mb (153,676,602–159,302,377) region in the CS reference genome (Figure 2C).

### Development of Tightly Linked Markers to *LrLC10* (*Lr13*)

We used those co-dominant flanking markers, *CAUT163* and *Lseq22*, to identify recombinants in the 984 homozygous, susceptible F_2_ plants. Thirty-two recombinant plants were identified and then used for fine mapping of *LrLC10*.

**FIGURE 3:**
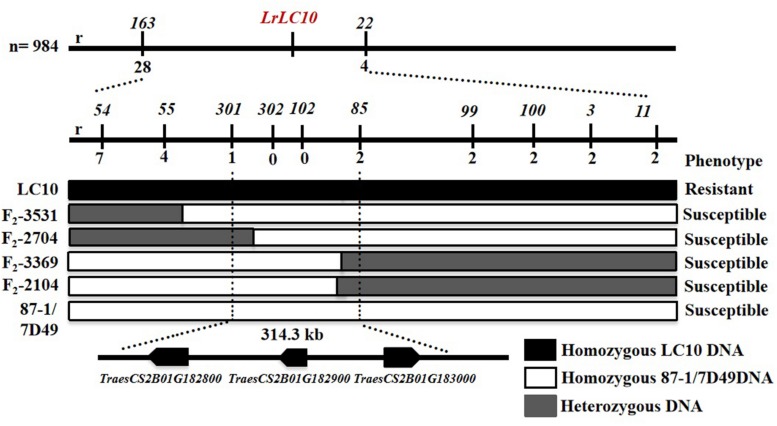
Fine mapping of *LrLC10* and three annotated genes localize in the mapping interval on Chinese Spring reference genome. Phenotypes and genotypes of four F_2_ recombinants including (F_2_-3531, F_2_-2704, F_2_-3369, F_2_-2104) are showed. The name and phenotype of F_2_ individuals were labeled in the left and right, respectively. Black, white and gray blocks present genomic region of LC10, 87-1/7D49 and heterozygous, respectively. r indicated the number of recombinants.

Based on the parents’ re-sequencing data that corresponded to the 5.7 Mb interval of the CS RefSeq v1.0, we designed 80 indel primers and a KASP marker. They were tested on 3 parental lines and 10 markers were polymorphic between the parents and used to finely map *LrLC10* (Figure 3).

**FIGURE 4:**
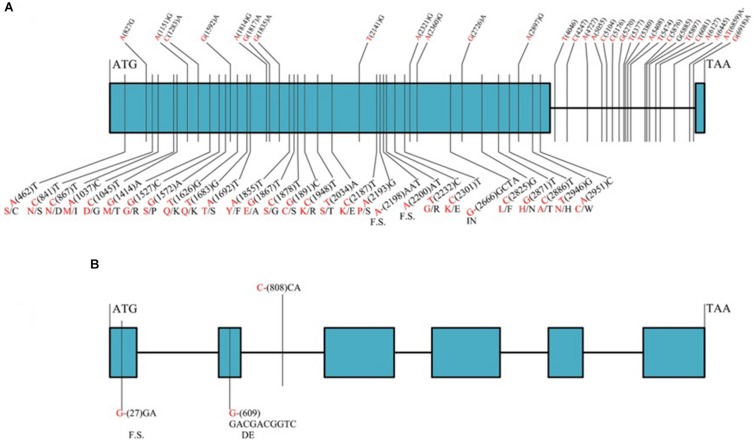
Structure of annotated genes [*TraesCS2B01G182800* and *TraesCS2B01G183000* **(A,B)**] showing the nucleotide and amino acid sequences polymorphism between resistant and susceptible parents. Introns, exons are shown in lines, blue boxes. Red and black fonts represent resistant and susceptible parents, respectively. The numbers in bracket represent the positions of nucleotide sequences relative to ATG. F.S. represents frame shift, - indicates nucleotide deletion, IN represents amino acid insertion, DE represents amino acid deletion.

Among the 32 recombinant plants, 28 showed recombination between marker *CAUT163* and *LrLC10*, while 4 recombination events were detected between marker *Lseq22* and *LrLC10*. We used the 10 polymorphic markers located between the flanking markers to examine these recombinants and found that the closest flanking markers were *Lseq301* (with 1 recombination event) and *Lseq85* (with 2 recombination events) and markers *Lseq302* and *Lseq102* co-segregated with *LrLC10* (Figure 3). These results suggest that *LrLC10* locus is located in a 314.3-kb region between markers *Lseq85* and *Lseq301* (157,688,415–158,002,717) in the CS RefSeq v1.0 (Figure 3). Markers *Lseq85* and *Lseq301* were designed based on the 5 bp and 6 bp deletions, respectively, in Liaochun10 as compared to 7D49. The KASP marker *Lseq302* was based on the SNP (A/T) detected in exon 2 of *TraesCS2B01G182800* between Liaochun10 and 7D49 (Figure 4A and Table 3), while *Lseq102* was developed based on a 9-bp deletion in the *TraesCS2B01G183000* coding region between Liaochun10 and 7D49 (Figure 4B and Table 3).

**TABLE 3:** Sequence comparison of the annotated genes.

Gene-ID	Position on reference	Region of the gene	Position in the gene	Sequence variants (LC10/7D49)	Protein variants (LC10/7D49)
*TraesCS2B01G182800*	157695820	Exon2	462	A/T	S/C
	157695455	Exon2	827	A/G	–
	157695441	Exon2	841	C/T	N/S
	157695415	Exon2	867	C/T	N/D
	157695245	Exon2	1037	A/C	M/I
	157695237	Exon2	1045	C/T	D/G
	157695131	Exon2	1151	A/G	–
	157694999	Exon2	1283	C/A	–
	157694868	Exon2	1414	G/A	M/T
	157694755	Exon2	1527	G/C	G/R
	157694710	Exon2	1572	G/A	S/P
	157694690	Exon2	1592	G/A	–
	157694656	Exon2	1626	T/G	Q/K
	157694599	Exon2	1683	T/G	Q/K
	157694590	Exon2	1692	A/T	T/S
	157694468	Exon2	1814	A/G	–
	157694465	Exon2	1817	G/A	–
	157694447	Exon2	1835	G/A	–
	157694427	Exon2	1855	A/T	Y/F
	157694415	Exon2	1867	G/T	E/A
	157694404	Exon2	1878	C/T	S/G
	157694391	Exon2	1891	G/C	C/S
	157694334	Exon2	1948	C/T	K/R
	157694248	Exon2	2034	T/A	S/T
	157694141	Exon2	2141	T/G	–
	157694095	Exon2	2187	C/T	K/E
	157694089	Exon2	2193	A/G	P/S
	157694084	Exon2	2198	A-/AAT	Frame shift
	157694082	Exon2	2200	A-/AT	Frame shift
	157694050	Exon2	2232	T/C	G/R
	157693981	Exon2	2301	C/T	K/E
	157693961	Exon2	2321	A/G	–
	157693922	Exon2	2360	A/G	–
	157693616	Exon2	2666	G-/GCTA	Insertion
	157693556	Exon2	2726	G/A	–
	157693457	Exon2	2825	C/G	L/F
	157693411	Exon2	2871	G/T	H/N
	157693396	Exon2	2886	C/T	A/T
	157693385	Exon2	2897	A/G	–
	157693336	Exon2	2946	T/G	N/H
	157693331	Exon2	2951	A/C	C/W
	157692236	Intron2	4046	T/C	–
	157692035	Intron2	4247	C/T	–
	157691555	Intron2	4727	A/G	–
	157691227	Intron2	5055	A/G	–
	157691178	Intron2	5104	C/T	–
	157691106	Intron2	5176	C/T	–
	157691012	Intron2	5270	G/A	–
	157690905	Intron2	5377	T/C	–
	157690902	Intron2	5380	T/C	–
	157690874	Intron2	5408	A/G	–
	157690808	Intron2	5474	T/C	–
	157690406	Intron2	5876	C/T	–
	157690397	Intron2	5885	G/A	–
	157690385	Intron2	5897	T/C	–
	157690201	Intron2	6081	C/T	–
	157690155	Intron2	6127	A/G	–
	157689837	Intron2	6445	A/G	–
	157689423	Intron2	6859	AT/A-	–
	157689364	Intron2	6918	G/A	–
*TraesCS2B01G183000*	157755306	Exon1	27	G-/GA	Frame shift
	157755888	Exon2	609	G-/GACGACGGTC	Deletion
	157756087	Intron2	808	C-/CA	–

*Position in the gene represents the positions of nucleotide sequences relative to ATG.*

### Validation of *LrLC10*-Co-segregating Markers for Marker-Assisted Selection

**FIGURE 5:**
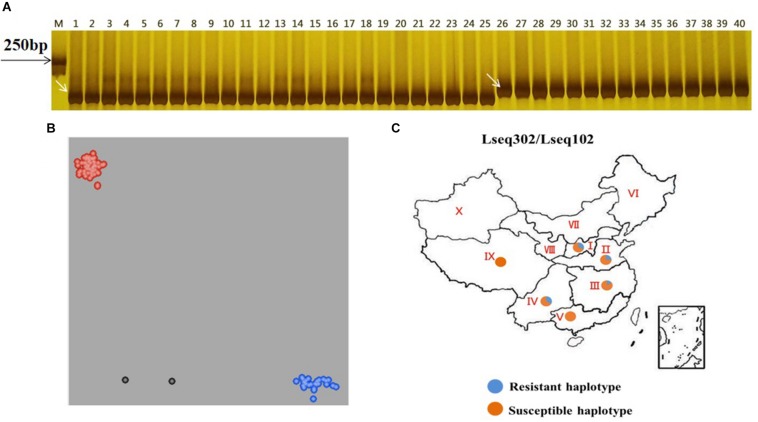
Validation of *Lr13* diagnostic markers **(A,B)** and haplotype distribution of *Lseq302* and *Lseq102* in 524 common wheat cultivars/landraces from China **(C)**. A co-segregated marker *Lseq102* genotyped several *Lr13* carriers and susceptible accessions in PAGE gel. The number 1 and 24 present Liaochun10 and 87-1, respectively, 2–23, 25–31 indicate *Lr13* carriers and susceptible lines, respectively. M: Marker; B plots of KASP marker *Lseq302* tested 23 *Lr13* carriers (blue dots), 10 susceptible lines (red dots) and other cultivars with unknown leaf rust resistance. Black dots are water controls; C I, north China winter wheat region; II, Yellow and Huai River valley winter wheat region; III, middle and lower Yangtze River valley winter wheat region; IV, south-western winter wheat region; V, south China winter wheat region; VI, north-eastern spring wheat region; VII, northern spring wheat region; VIII, north-western spring wheat region; IX, Qinghai–Tibet spring–winter wheat region; X, Xinjiang winter–spring wheat region.

We wanted to test if these co-segregating markers (*Lseq302* and *Lseq102*) could be used for marker-assisted selection of *LrLC10* in different backgrounds. We tested 25 wheat leaf rust resistant accessions and 10 susceptible cultivars with those 2 markers to evaluate their utility. Twenty-three of the resistant accessions had the same marker genotypes as Liaochun10 and all the susceptible cultivars’ genotypes were identical to 87-1 and 7D49 (Figures 5A,B and Table 1). Moreover, we found that the F_1_ plants of the crosses of those 14 of the 23 resistant accessions with Xuezao, which was proved to carry the hybrid necrosis gene *Ne1* (unpublished results), showed progressive necrosis (Table 1). According to Zhang et al. (2016), *Lr13* and *Ne2m* are the same gene; so by inference, the F_1_ plants’ phenotypes indicate that those 14 cultivars have leaf rust resistance gene *Lr13*. These results suggest that markers *Lseq302* and *Lseq102* can be used to identify *Lr13*.

To evaluate the distribution of Lseq302-L and Lseq102-L (the marker alleles of *Lseq302*/*Lseq102* in Liaochun10) in China, a panel of 524 common wheat accessions/landraces from China was tested with these markers. Lseq302-L and Lseq102-L always co-existed in all the cultivars, forming a specific haplotype block. The haplotype of Lseq302-L/Lseq102-L was present in various frequencies in 4 of 10 agro-ecological production zones: I, North China winter wheat region (30.76%); II, Yellow and Huai River valleys winter wheat region (24.56%); III, middle and lower Yangtze River valley winter wheat region (14.81%); and V, South China winter wheat region (33.33%) (Figure 5C and Table 4).

**TABLE 4:** Percentage of different alleles in Chinese accessions.

		*Lseq302*				*Lseq102*			
		*Lseq302-A*		*Lseq302-B*		*Lseq102-A*		*Lseq102-B*	
Agroecological region	No. of accessions	No. of accessions	Percentage of the allele	No. of accessions	Percentage of the allele	No. of accessions	Percentage of the allele	No. of accessions	Percentage of the allele
I	89	27	0.3	62	0.7	27	0.3	62	0.7
II	388	94	0.24	294	0.76	94	0.24	294	0.76
III	27	4	0.15	23	0.85	4	0.15	23	0.85
V	15	5	0.33	10	0.67	5	0.33	10	0.67
IX	4	0	0	4	1	0	0	4	1
Unkown	1	0	0	1	1	0	0	1	1
Total	524	130	0.25	394	0.75	130	0.25	394	0.75

*I, Northern winter wheat region; II, Yellow and Huai River valley winter wheat region; III, low and middle Yangtze River valley winter wheat region; V, south-western winter wheat region; IX, Qinghai–Tibet spring-winter wheat region.*

## Discussion

Seven leaf rust resistance genes (*Lr13*, *Lr16*, *Lr23*, *Lr35*, *Lr48*, *Lr73*, and *LrZH22*) had been mapped on wheat chromosome 2BS (Seyfarth et al., 1999; Park et al., 2014; Nsabiyera et al., 2016; Wang et al., 2016; Zhang et al., 2016; Chhetri et al., 2017; Kassa et al., 2017). We found that *LrLC10* is located in the region 157,688,415–158,002,717 bp (Figure 3) (2BS1-0.53-0.75) on the reference sequence of Chinese Spring (RefSeq v1.0). Of those 7 *Lr* genes, only *Lr13* and *LrZH22* were reported to be located in the same region as *LrLC10* (Wang et al., 2016; Zhang et al., 2016). *LrZH22* confers resistance to leaf rust at both the seedling and adult stages (Wang et al., 2016), whereas resistance gained from *LrLC10* is effective only after the four-leaf stage. Up to now, the relationship between *LrLC10* and *Lr13* was unknown, but we confirmed that *LrLC10* is *Lr13*. The pedigree of Liaochun10 is 1048 (Ke71F_4_370-10/Mexipak66//UP301) × Liao70181-2 (Liaochun6/Jinghong1) and the parents of Liaochun6 are Frontana and Liaochun1 (He et al., 2001). Since the leaf rust resistance gene in Frontana and UP301 is *Lr13* (Singh and Gupta, 1991; Pathan and Park, 2006), the leaf rust resistance gene in Liaochun10 may be derived from the Frontana or UP301 parent.

In the process of fine mapping *LrLC10*, enough polymorphic markers were found to narrow down the genetic interval covering the targeted gene. We accomplished this by re-sequencing the parental lines and developing indel and SNP markers based on sequence information in the targeted region. Four indel markers revealed polymorphisms and localized *LrLC10* gene in a 1.65 cM genetic interval, which corresponded to a 5.7 Mb interval on the Chinese Spring reference genomic sequence (Figures 2B,C). Based on sequence diversities between the parental lines on the candidate interval, we designed 9 indel markers and a KASP marker and used these to test the 32 recombinants that we identified using *LrLC10*-flanking markers *CAUT163* and *Lseq22* derived from 984 homozygous, susceptible F_2_ individuals. *LrLC10* was finally delimited into a 314.3-kb genomic interval on the Chinese Spring reference sequence v1.0 by markers *Lseq301* and *Lseq85* (Figure 3). This mapping of *LrLC10* demonstrated our methods efficiently developed molecular markers based from the re-sequencing data of the parents.

*Lr13* is one of the most widely distributed leaf rust resistance genes in wheat (McIntosh et al., 1995), but it has become ineffective in some regions, such as Mexico and South America (Singh and Rajaram, 1992). However, it is effective in combination with other resistance genes, such as *Lr3ka*, *Lr34*, and *Lr16* (Kolmer, 1992). Therefore, we need diagnostic molecular markers for *Lr13* to facilitate selection or stacking it with other *Lr* genes. In our study, KASP marker *Lseq302* and indel marker *Lseq102* co-segregated with *Lr13* and were effective in diverse wheat backgrounds (Figures 5A,B, Table 1, and Supplementary Table S1). Therefore, these diagnostic markers may be used for efficient marker-assisted selection of *Lr13*, thus enabling researchers to either pyramid it with other adult plant resistant genes to achieve durable leaf rust resistance or stack it with stripe rust, stem rust, and powdery mildew resistance genes (e.g., *Yr27*, *Sr40*, and *pm42*) on chromosome 2BS, to create multi-resistance accessions (McDonald et al., 2004; Hua et al., 2009; Wu et al., 2009).

To date, several leaf rust resistance genes have been isolated in wheat: *Lr1*, *Lr10*, *Lr21*, *Lr34*, *Lr67*, and *Lr22a* (Feuillet et al., 2003; Huang et al., 2003; Cloutier et al., 2007; Krattinger et al., 2009; Moore et al., 2015; Thind et al., 2017). Among those genes, *Lr34* encodes an ATP-binding cassette transporter that carries resistance-related metabolites that affect the growth of pathogenic bacteria (Krattinger et al., 2009). *Lr67* encodes a hexose transporter, LR67res, that through heterodimerization with the *Lr67*- susceptible functional transporter LR67sus, exerted a dominant-negative effect that restricted the growth of multiple biotrophic pathogens by reducing their glucose uptake (Moore et al., 2015). *Lr34* and *Lr67* provide wheat with resistance to multiple fungal pathogens. The others are typical resistance genes with nucleotide-binding site leucine-rich repeat (NBS-LRR) domains (Feuillet et al., 2003; Huang et al., 2003; Cloutier et al., 2007; Thind et al., 2017). However, *LrLC10* (*Lr13*) was shown to be a race-specific resistance gene (Dyck et al., 1966), thus it most likely has the R-gene structure of NLR.

In this study, we delimited *LrLC10* to a 314.3 kb region on short arm of chromosome 2B in Chinese Spring reference genome sequence (RefSeq v1.0). Three high confidence genes (*TraesCS2B01G182800*, *TraesCS2B01G182900*, and *TraesCS2B01G183000*) have different functions based on the annotation of Chinese Spring reference genome^7^, which encode a typical NBS-LRR protein, Ribonuclease, and an F-box domain containing Leucine-rich repeats protein were located in this region. DNA sequence comparison showed that the parents did not differ in *TraesCS2B01G182900*. Compared to 7D49, Liaochun10 had a 9 bp deletion in its *TraesCS2B01G183000* coding region, resulting in a deletion of three amino acids, and another 1-bp deletion that led to a translational frame shift (Figure 4B and Table 3). In *TraesCS2B01G182800*, we found many sequence variations, including 56 SNPs and 4 indels, between Liaochun10 and its rust-susceptible parent. Among them, we found 18 SNPs and one indel in the intron region, and 38 SNPs and three indels in exon 2. Among those 38 SNPs, 26 caused amino acid substitutions, and two of the indels led to a translational frame shift. A 3-bp insertion in 7D49 resulted in an amino acid insertion that was not found in Liaochun10 (Figure 4A and Table 3). Based on the *TraesCS2B01G182800* and *TraesCS2B01G183000* sequence polymorphisms between the parental lines, we developed the markers *Lseq102* and *Lseq302* and found that they co-segregated with *LrLC10* (Figure 3). In total, our results suggest that *LrLC10* (*Lr13*) might be one of those two annotated genes. However, there is still a chance that the sequence corresponding to *LrLC10* is absent in the CS genomic sequence. Therefore, our analysis of re-sequencing data based on the wheat reference genome sequence is not enough to be absolutely certain of its identity. Because of this, a library of the resistant parent must be constructed so that a physical map would enable the cloning of *LrLC10*. Recently, some alternative methods (e.g., MutRenSeq, TACCA, and MutChromSeq) have been used to clone wheat disease resistance genes (Steuernagel et al., 2016; Sánchez-Martín et al., 2016; Thind et al., 2017) and may possibly be used to clone *LrLC10*. After all, the fine genetic map and co-segregating markers developed in our present study may aid the map-based cloning and the marker-assisted selection of *LrLC10* (*Lr13*).

## Data Availability Statement

The datasets generated for this study can be found in the European Variation Archive (EVA) using accession number PRJEB37197.

## Author Contributions

LQ and CX conceived the project. LQ, HW, WW, YuL, and JMu performed the experiments. MG and YiL assisted in revising the manuscript. WG analyzed the re-sequencing data. JMa, ZH, and QS provided materials. LQ wrote the manuscript. CX revised the manuscript.

## Conflict of Interest

The authors declare that the research was conducted in the absence of any commercial or financial relationships that could be construed as a potential conflict of interest.

## Footnotes

1 https://shigen.nig.ac.jp/wheat/komugi/

2 https://maswheat.ucdavis.edu/protocols/index.htm

3 https://www.wheatgenome.org/

4 https://www.wheatgenome.org/

5 http://bioinfo.ut.ee/primer3-0.4.0

6 http://www.polymarker.info/

7 https://urgi.versailles.inra.fr/download/iwgsc/IWGSC_RefSeq_Annotations/v1.0/

## References

[B1] Anand D. Saini R. G. Gupta A. K. (1991). Linkage distance between the wheat leaf rust resistance gene *Lr13* and a gene for hybrid necrosis *Ne2m*. *J. Genet. Breed.* 45 245–246. 26660463

[B2] Bansal U. K. Hayden M. J. Venkata B. P. Khanna R. Saini R. G. Bariana H. S. (2008). Genetic mapping of adult plant leaf rust resistance genes *Lr48* and *Lr49* in common wheat. *Theor. Appl. Genet.* 117 307–312. 10.1007/s00122-008-0775-6 18542911

[B3] Bariana H. S. Brown G. N. Bansal U. K. Miah H. Standen G. E. Lu M. (2007). Breeding triple rust resistant wheat cultivars for Australia using conventional and marker-assisted selection technologies. *Aust. J. Agric. Res.* 58 576–587. 10.1071/AR07124

[B4] Chai L. Chen Z. Bian R. Zhai H. Cheng X. Peng H. (2018). Dissection of two quantitative trait loci with pleiotropic effects on plant height and spike length linked in coupling phase on the short arm of chromosome 2D of common wheat (*Triticum aestivum* L.). *Theor. Appl. Genet.* 131 2621–2637. 10.1007/s00122-018-3177-4 30267114

[B5] Chen J. W. Wang L. Pang X. F. Pan Q. H. (2006). Genetic analysis and fine mapping of a rice brown planthopper (Nilaparvata lugens Stål) resistance gene *bph19*(t). *Mol. Genet. Genomics* 275 321–329. 10.1007/s00438-005-0088-2 16395578

[B6] Chhetri M. Bariana H. Wong D. Sohail Y. Hayden M. Bansal U. (2017). Development of robust molecular markers for marker-assisted selection of leaf rust resistance gene *Lr23* in common and durum wheat breeding programs. *Mol. Breed.* 37:21 10.1007/s11032-017-0628-6

[B7] Clavijo B. J. Venturini L. Schudoma C. Accinelli G. G. Kaithakottil G. Wright J. (2017). An improved assembly and annotation of the allohexaploid wheat genome identifies complete families of agronomic genes and provides genomic evidence for chromosomal translocations. *Genome Res.* 27 885–896. 10.1101/gr.217117.116 28420692 PMC5411782

[B8] Cloutier S. McCallum B. D. Loutre C. Banks T. W. Wicker T. Feuillet C. (2007). Leaf rust resistance gene *Lr1*, isolated from bread wheat (*Triticum aestivum* L.) is a member of the large psr567 gene family. *Plant Mol. Biol.* 65, 93–106. 10.1007/s11103-007-9201-8 17611798

[B9] Dong J. G. (2001). *Agricultural Plant Pathology.* Beijing: China Agriculture Press.

[B10] Dou B. Hou B. Xu H. Lou X. Chi X. Yang J. (2009). Efficient mapping of a female sterile gene in wheat (*Triticum aestivum* L.). *Genet. Res.* 91 337–343. 10.1017/S0016672309990218 19922697

[B11] Dyck P. L. Samborski D. J. Anderson R. G. (1966). Inheritance of adult-plant leaf rust resistance derived from the common wheat varieties Exchange and Frontana. *Can. J. Genet. Cytol.* 8 665–671. 10.1139/g66-082

[B12] FAOSTAT (2015). *FAO statistical Pocketbook 2015: World Food and Agriculture.* Rome: Food and Agriculture Organization of the United Nations.

[B13] Feng J. Chen G. Wei Y. Liu Y. Jiang Q. Li W. (2015). Identification and mapping stripe rust resistance gene *YrLM168a* using extreme individuals and recessive phenotype class in a complicate genetic background. *Mol. Genet. Genomics* 290 2271–2278. 10.1007/s00438-015-1077-8 26113523

[B14] Feuillet C. Travella S. Stein N. Albar L. Nublat A. Keller B. (2003). Map-based isolation of the leaf rust disease resistance gene *Lr10* from the hexaploid wheat (*Triticum aestivum* L.) *genome*. *Proc. Natl. Acad. Sci. U.S.A.* 100 15253–15258. 10.1073/pnas.2435133100 14645721 PMC299976

[B15] He Z. H. Rajaram S. Huang G. Z. (2001). *A History of Wheat Breeding in China.* Mexico City: CIMMYT.

[B16] Hua W. Liu Z. Zhu J. Xie C. Yang T. Zhou Y. (2009). Identification and genetic mapping of *pm42*, a new recessive wheat powdery mildew resistance gene derived from wild emmer (*Triticum turgidum var*. dicoccoides). *Theor. Appl. Genet.* 119 223–230. 10.1007/s00122-009-1031-4 19407985

[B17] Huang L. Brooks S. A. Li W. Fellers J. P. Trick H. N. Gill B. S. (2003). Map-Based cloning of leaf rust resistance gene *Lr21* from the large and polyploid genome of bread wheat. *Genetics* 164 655–664. 12807786 10.1093/genetics/164.2.655 PMC1462593

[B18] Jiang B. Liu T. Li H. Han H. Li L. Zhang J. (2018). Physical mapping of a novel locus conferring leaf rust resistance on the long arm of *Agropyron cristatum* Chromosome 2P. *Front. Plant Sci.* 9:817. 10.3389/fpls.2018.00817 29971077 PMC6018490

[B19] Kassa M. T. You F. M. Hiebert C. W. Pozniak C. J. Fobert P. R. Sharpe A. G. (2017). Highly predictive SNP markers for efficient selection of the wheat leaf rust resistance gene *Lr16*. *BMC Plant Biol.* 17:45. 10.1186/s12870-017-0993-7 28202046 PMC5311853

[B20] Kiswara G. Lee J. H. Hur Y. J. Cho J. H. Lee J. Y. Kim S. Y. (2014). Genetic analysis and molecular mapping of low amylose gene *du12*(t) in rice (*Oryza sativa* L.). *Theor. Appl. Genet.* 127 51–57. 10.1007/s00122-013-2200-z 24114051

[B21] Kolmer J. A. (1992). Enhanced leaf rust resistance in wheat conditioned by resistance gene pairs with *Lr13*. *Euphytica* 61 123–130. 10.1007/BF00026802

[B22] Kolmer J. A. Su Z. Bernardo A. Bai G. Chao S. (2018). Mapping and characterization of the new adult plant leaf rust resistance gene *Lr77* derived from Santa Fe winter wheat. *Theor. Appl. Genet.* 131 1553–1560. 10.1007/s00122-018-3097-3 29696297

[B23] Kosambi D. D. (1943). The estimation of map distance from recombination values. *Ann. Eugen.* 12 172–175. 10.1111/j.1469-1809.1943.tb02321.x

[B24] Krattinger S. G. Lagudah E. S. Spielmeyer W. Singh R. P. Huerta-Espino J. McFadden H. (2009). A putative ABC transporter confers durable resistance to multiple fungal pathogens in wheat. *Science* 323 1360–1363. 10.1126/science.1166453 19229000

[B25] Liu R. Meng J. (2003). MapDraw: a microsoft excel macro for drawing genetic linkage maps based on given genetic linkage data. *Hereditas* 25 317–321. 10.16288/j.yczz.2003.03.017 15639879

[B26] Lv X. Tang H. Geng M. Mi Y. Li Y. Li F. (2017). Comparative genomics analysis of leaf rust resistance gene *LrLC10* in common wheat cultivar Liaochun10. *J. China Agric. Univ.* 22 01–09.

[B27] McDonald D. B. McIntosh R. A. Wellings C. R. Singh R. P. Nelson J. C. (2004). Cytogenetical studies in wheat XIX. location and linkage studies on gene *Yr27* for resistance to stripe (yellow) rust. *Euphytica* 136 239–248. 10.1023/B:EUPH.0000032709.59324.45

[B28] McIntosh R. A. Dubcovsky J. Rogers W. J. Morris C. Xia X. (2017). *Catalogue of Gene Symbols for Wheat: 2017 Supplement.* Avaliable at: https://shigen.nig.ac.jp/wheat/komugi/genes/macgene/supplement2017.pdf. (accessed September 20, 2017).

[B29] McIntosh R. A. Wellings C. R. Park R. F. (1995). *Wheat Rusts: An Atlas of Resistance Genes.* Melbourne: Csiro Publishing.

[B30] Mei M. H. Dai X. K. Xu C. G. Zhang Q. (1999). Mapping and genetic analysis of the genes for photoperiod-sensitive genic male sterility in rice using the original mutant Nongken 58S. *Crop Sci.* 39 1711–1715. 10.2135/cropsci1999.3961711x 15912317

[B31] Michelmore R. W. Paran I. Kesseli R. V. (1991). Identification of markers linked to disease-resistance genes by bulked segregant analysis: a rapid method to detect markers in specific genomic regions by using segregating populations. *Proc. Natl. Acad. Sci. U.S.A.* 88 9828–9832. 10.1073/pnas.88.21.9828 1682921 PMC52814

[B32] Moore J. W. Herrera-Foessel S. Lan C. Schnippenkoetter W. Ayliffe M. Huerta-Espino J. (2015). A recently evolved hexose transporter variant confers resistance to multiple pathogens in wheat. *Nat. Genet.* 47 1494–1498. 10.1038/ng.3439 26551671

[B33] Narang D. Kaur S. Steuernagel B. Ghosh S. Dhillon R. Bansal M. (2019). Fine mapping of *Aegilops peregrina* co-segregating leaf and stripe rust resistance genes to distal-most end of 5DS. *Theor. Appl. Genet.* 132 1473–1485. 10.1007/s00122-019-03293-5 30706082

[B34] Nsabiyera V. Qureshi N. Bariana H. S. Wong D. Forrest K. L. Hayden M. J. (2016). Molecular markers for adult plant leaf rust resistance gene *Lr48* in wheat. *Mol. Breed.* 36:65 10.1007/s11032-016-0488-5

[B35] Park R. F. Mohler V. Nazari K. Singh D. (2014). Characterisation and mapping of gene *Lr73* conferring seedling resistance to *Puccinia triticina* in common wheat. *Theor. Appl. Genet.* 127 2041–2049. 10.1007/s00122-014-2359-y 25116148

[B36] Pathan A. K. Park R. F. (2006). Evaluation of seedling and adult plant resistance to leaf rust in European wheat cultivars. *Euphytica* 149 327–342. 10.1007/s10681-005-9081-4

[B37] Qureshi N. Bariana H. Kumran V. V. Muruga S. Forrest K. L. Hayden M. J. (2018). A new leaf rust resistance gene *Lr79* mapped in chromosome 3BL from the durum wheat landrace Aus26582. *Theor. Appl. Genet.* 131 1091–1098. 10.1007/s00122-018-3060-3 29396589

[B38] Ren X. Liu T. Liu B. Gao L. Chen W. Q. (2015). Postulation of seedling leaf rust resistance genes in 84 Chinese winter wheat cultivars. *J. Integrat. Agric.* 14 1992–2001. 10.1016/S2095-3119(14)61002-9

[B39] Roelfs A. P. (1988). *Resistance to Leaf and Stem Rusts in Wheat. Breeding Strategies for Resistance to the Rusts of Wheat.* Mexico: CIMMYT.

[B40] Roelfs A. P. Singh R. P. Saari E. E. (1992). *Rust Diseases of Wheat: Concepts and Methods of Disease Management.* Mexico: CIMMYT.

[B41] Sánchez-Martín J. Steuernagel B. Ghosh S. Herren G. Hurni S. Adamski N. (2016). Rapid gene isolation in barley and wheat by mutant chromosome sequencing. *Genome Biol.* 17:221. 10.1186/s13059-016-1082-1 27795210 PMC5087116

[B42] Seyfarth R. Feuillet C. Schachermayr G. Winzeler M. Keller B. (1999). Development of a molecular marker for the adult plant leaf rust resistance gene *Lr35* in wheat. *Theor. Appl. Genet.* 99 554–560. 10.1007/s001220051268 22665189

[B43] Singh A. Knox R. E. DePauw R. M. Singh A. K. Cuthbert R. D. Campbell H. L. (2013). Identification and mapping in spring wheat of genetic factors controlling stem rust resistance and the study of their epistatic interactions across multiple environments. *Theor. Appl. Genet.* 126 1951–1964. 10.1007/s00122-013-2109-6 23649649 PMC3723980

[B44] Singh R. P. Chen W. Q. He Z. H. (1999). Leaf rust resistance of spring, facultative, and winter wheat cultivars from china. *Plant Dis.* 83 644–651. 10.1094/PDIS.1999.83.7.644 30845616

[B45] Singh R. P. Gupta A. K. (1991). Genes for leaf rust resistance in Indian and Pakistani wheats tested with Mexican pathotypes of *Puccinia recondita* f. sp. tritici. *Euphytica* 57 27–36. 10.1007/BF00040475

[B46] Singh R. P. Rajaram S. (1992). Genetics of adult-plant resistance of leaf rust in ‘Frontana’ and three CIMMYT wheats. *Genome* 35 24–31. 10.1139/g92-004

[B47] Steuernagel B. Periyannan S. K. Hernández-Pinzón I. Witek K. Rouse M. N. Yu G. (2016). Rapid cloning of disease-resistance genes in plants using mutagenesis and sequence capture. *Nat. Biotechnol.* 34 652–655. 10.1038/nbt.3543 27111722

[B48] Thind A. K. Wicker T. Šimková H. Fossati D. Moullet O. Brabant C. (2017). Rapid cloning of genes in hexaploid wheat using cultivar-specific long-range chromosome assembly. *Nat. Biotechnol.* 35 793–796. 10.1038/nbt.3877 28504667

[B49] Varshney R. K. Terauchi R. McCouch S. R. (2014). Harvesting the promising fruits of genomics: applying genome sequencing technologies to crop breeding. *PLoS Biol.* 12:e1001883. 10.1371/journal.pbio.1001883 24914810 PMC4051599

[B50] Wang C. Yin G. Xia X. He Z. Zhang P. Yao Z. (2016). Molecular mapping of a new temperature-sensitive gene *LrZH22* for leaf rust resistance in Chinese wheat cultivar Zhoumai 22. *Mol. Breed.* 36:18 10.1007/s11032-016-0437-3

[B51] WAP (2017). “World agricultural production,” in *Circular Series, WAP* 01–17, Washington, DC: United States Department of Agriculture-Foreign Agricultural Service.

[B52] Wu J. Liu S. Wang Q. Zeng Q. Mu J. Huang S. (2018a). Rapid identification of an adult plant stripe rust resistance gene in hexaploid wheat by high-throughput SNP array genotyping of pooled extremes. *Theor. Appl. Genet.* 131 43–58. 10.1007/s00122-017-2984-3 28965125

[B53] Wu J. Zeng Q. Wang Q. Liu S. Yu S. Mu J. (2018b). SNP-based pool genotyping and haplotype analysis accelerate fine-mapping of the wheat genomic region containing stripe rust resistance gene *Yr26*. *Theor. Appl. Genet.* 131 1481–1496. 10.1007/s00122-018-3092-8 29666883

[B54] Wu P. Hu J. Zou J. Qiu D. Qu Y. Li Y. (2019). Fine mapping of the wheat powdery mildew resistance gene *Pm52* using comparative genomics analysis and the Chinese Spring reference genomic sequence. *Theor. Appl. Genet.* 132 1451–1461. 10.1007/s00122-019-03291-7 30719526

[B55] Wu S. Pumphrey M. Bai G. (2009). Molecular mapping of stem-rust-resistance gene in wheat. *Crop Sci.* 49:1681 10.2135/cropsci2008.11.0666

[B56] Xu Y. Li P. Zou C. Lu Y. Xie C. Zhang X. (2017). Enhancing genetic gain in the era of molecular breeding. *J. Exp. Bot.* 68 2641–2666. 10.1093/jxb/erx135 28830098

[B57] Yao F. Xu C. Yu S. Li J. Gao Y. Li X. (1997). Mapping and genetic analysis of two fertility restorer loci in the wild-abortive cytoplasmic male sterility system of rice (*Oryza sativa* L.). *Euphytica* 98 183–187. 10.1023/A:1003165116059

[B58] Yuan J. Chen W. (2011). Estimate on the effectiveness of main resistant genes for leaf rust in Chinese Wheat. *J. Triticeae Crops* 31 994–999.

[B59] Yuan J. Liu T. Chen W. (2007). Postulation of leaf rust resistance genes in 47 new wheat cultivars (lines) at seedling stage cultivars (lines) at seedling stage. *Sci. Agric. Sinica* 40 1925–1935.

[B60] Zhang P. Gebrewahid T. W. Zhou Y. Li Q. Li Z. Liu D. (2019). Seedling and adult plant resistance to leaf rust in 46 Chinese bread wheat landraces and 39 wheat lines with known *Lr* genes. *J. Integrat. Agric.* 18 1014–1023. 10.1016/S2095-3119(19)62575-X

[B61] Zhang P. Hiebert C. W. McIntosh R. A. McCallum B. D. Thomas J. B. Hoxha S. (2016). The relationship of leaf rust resistance gene *Lr13* and hybrid necrosis gene *Ne2m* on wheat chromosome 2BS. *Theor. Appl. Genet.* 129 485–493. 10.1007/s00122-015-2642-6 26660463

[B62] Zhang Q. Shen B. Z. Dai X. K. Mei M. H. Maroof M. A. S. Li Z. (1994). Using bulked extremes and recessive class to map genes for photoperiod-sensitive genic male sterility in rice. *Proc. Natl. Acad. Sci. U.S.A* 91 8675–8679. 10.1073/pnas.91.18.8675 7915844 PMC44669

[B63] Zhou H. Xia X. He Z. Li X. Wang C. Li Z. (2013). Molecular mapping of leaf rust resistance gene *LrNJ97* in Chinese wheat line Neijiang 977671. *Theor. Appl. Genet.* 126 2141–2147. 10.1007/s00122-013-2124-7 23689746

[B64] Zou S. Wang H. Li Y. Kong Z. Tang D. (2018). The NB-LRR gene *Pm60* confers powdery mildew resistance in wheat. *New Phytol.* 218 298–309. 10.1111/nph.14964 29281751

